# Outcomes of transitional care programs on adolescent chronic inflammatory systemic diseases: systematic review and meta-analyses

**DOI:** 10.1186/s12969-022-00670-1

**Published:** 2022-02-17

**Authors:** Fernando García-Rodríguez, Karina Raygoza-Cortez, Lesli Moreno-Hernandez, Rodrigo García-Pérez, Leticia Elizabeth Garza Lopez, Ana Cecilia Arana-Guajardo, Joel Omar Jáquez-Quintana, Ana Victoria Villarreal-Treviño, Manuel Enrique de la O-Cavazos, Nadina Rubio-Pérez

**Affiliations:** 1 Department of Pediatrics, School of Medicine and University Hospital “Dr. José E. González”, Universidad Autónoma de Nuevo León, Av Madero Y Gonzalitos S/N, Col. Mitras Centro, 64460 Monterrey, Mexico; 2 Plataforma INVEST Medicina UANL-KER Unit Mayo Clinic (KER Unit Mexico), Universidad Autónoma de Nuevo León, Monterrey, 64460 México; 3 Servicio de Reumatología, Instituto de Medicina Interna. Escuela Nacional de Medicina Sistema Tec Salud, Monterrey, Mexico; 4 Gastroenterology Service and Department of Internal Medicine, School of Medicine and University Hospital “Dr. José E. González”, Universidad Autónoma de Nuevo León, Av Madero Y Gonzalitos S/N, Col. Mitras Centro, 64460 Monterrey, Mexico

**Keywords:** Transition program, Chronic conditions, Inflammatory bowel disease, Juvenile idiopathic arthritis, Systematic review

## Abstract

**Background:**

Patients with juvenile chronic inflammatory systemic diseases (jCID) are vulnerable to many circumstances when transitioning to adult-centered healthcare; this increases the burden of disease and worsen their quality of life.

**Methods:**

MEDLINE, Embase, Web of Science and Scopus were searched from inception to March 16^th^, 2021. We included observational, randomized controlled trials and quasi-experimental studies that evaluated a transitional care program for adolescents and young adults with jCIDs. We extracted information regarding health-related quality of life, disease activity, drop-out rates, clinical attendance rates, hospital admission rates, disease-related knowledge, surgeries performed, drug toxicity and satisfaction rates.

**Results:**

Fifteen studies met our inclusion criteria. The implementation of transition programs showed a reduction on hospital admission rates for those with transition program (OR 0.28; 95% CI 0.13 to 0.61; I 2 = 0%; *p* = 0.97), rates of surgeries performed (OR 0.26; 95% CI 0.12 to 0.59; I 2 = 0%; *p* = 0.50) and drop-out rates from the adult clinic (OR 0.23; 95% CI 0.12 to 0.46; I 2 = 0%; *p* = 0.88). No differences were found in other outcomes.

**Conclusion:**

The available body of evidence supports the implementation of transition programs as it could be a determining factor to prevent hospital admission rates, surgeries needed and adult clinic attendance rates.

**Supplementary Information:**

The online version contains supplementary material available at 10.1186/s12969-022-00670-1.

## Introduction

Adolescents and young adults with chronic inflammatory systemic diseases (jCID) transitioning from pediatric to adult services are vulnerable to multiple factors that increases the burden of disease [1–4]. Health care transition has been defined as the process of moving from a child to an adult model of health care with or without a transfer to a new clinician, with a preferred individualized process carried out by a multidisciplinary team of health care professionals [5].

The importance of these interventions during this period has been mentioned in numerous reports [6]. A successful transition has been associated with favorable outcomes in patients with inflammatory bowel disease (IBD) [7], type 1 diabetes [8], juvenile-onset rheumatic and musculoskeletal diseases (jRMD) [9] and youths with special needs [10]. Outcomes that have been most beneficial are those related to adherence to care, quality of life, experience of care, and service utilization and mortality [11]. Whereas a failed transition is linked to higher rates of treatment drop-outs and complications [12].

Despite that transition programs should help fill in the gaps in health care for patients living with jCID,(13) their implementation is not an universal practice yet. The high costs and necessary time to design and applicate them are the most important difficulties [14]. Prior et al. [15] developed a triple aim framework of transition measures including experience of care, population health and cost, but agreed that most of transition intervention studies report only one item in the framework and do so inconsistently. Besides, items such as health-related quality of life, disease activity, treatment adherence, and patient satisfaction are frequently missed.

The objective of this study is to systematically review and critically approach the available evidence regarding the outcomes of transition programs for patients with jCID.

## Methods

### Study design

This study adheres to the Preferred Reporting Items for Systematic Review and Meta-Analysis Protocols (PRISMA-P) statement. This review is registered on PROSPERO (CRD42021233777).

### Eligibility criteria

We included observational, randomized controlled trials (RCT) and quasi-experimental studies that mentioned the use of any transitional care program (TP) for adolescents and young adults (Age 11–25) with chronic inflammatory systemic diseases with at least one outcome of interest. Outcomes of interest included: (1) health related quality of life, as reported from a validated tool, (2) disease activity, as reported from a validated measure tool and/or treating physician, (3) drop-out rates during transition and on adult clinic (defined as a patient who did not attend any follow up appointment one year after the last appointment in the pediatric clinic or as defined by the author), (4) clinical attendance rates, (5) hospital admission rates, (6) surgeries performed, (7) satisfaction rate measured by any instrument (polls, descriptive, scales), (8) drug toxicity (as defined by the author) and (9) disease related knowledge. No date or language restrictions were applied.

### Search strategy and data management

An experienced librarian with input from the principal study investigators designed and conducted the search strategy, which was also revised and approved by all the investigators. The following electronic databases were searched from their time of inception to March 16^th^, 2021: MEDLINE, Embase, Web of Science and Scopus. We complemented the initial search strategy by consulting experts in the field, screening the reference lists from the eligible selected studies to identify any potentially relevant studies that may have been missed, and by searching for clinical trial registries to identify any unpublished or in-progress eligible studies. The full search strategy can be found in Supplementary Table 1. All search results were uploaded to EndNote X8 to avoid duplication. The resulting studies were uploaded to Distiller Systematic Review (DSR) for both, abstract and full-text screening.

### Study selection process

The study selection process took place in two phases. Through each phase of the review, four independent reviewers worked in duplicate to assess the eligibility of the studies. Chance adjusted inter-rater agreement was assessed using Kappa statistics. Prior to each phase, a pilot test was carried out to standardize the reviewers’ criteria. The pilot was repeated until a kappa index of > 0.70 was reached. Abstracts were then screened, when reviewers agreed, studies were moved to full-text screening or excluded. Abstracts with disagreements between reviewers were automatically considered for the full-text screening phase. Full-text articles where reviewers were not in agreement were discussed with a third reviewer until consensus was reached.

### Data collection process

Four independent reviewers, working in duplicate, collected data for all eligible articles using a web-based data extraction form. We gathered information regarding study setting, title, author information, funding, year of publication, baseline characteristics of patients (such as age, gender, medication, diagnosis and disease status at transfer, during transfer and after), disease and description of the transition program and outcomes of interest. Conflicts in this phase were resolved by consensus or arbitration by a third, experienced reviewer.

### Risk of bias in individual studies

Four independent reviewers, working in duplicate, performed the critical appraisal of the studies. RCTs were appraised using the Cochrane’s Risk of Bias tool 2.0 (RoB 2) [16]. We assessed the risk of bias in random sequence generation and allocation concealment (selection bias), blinding of participants and personnel (performance bias), blinding of the outcome assessment (detection bias), incomplete outcome data (attrition bias), and selective reporting (reporting bias). For observational studies involving an intervention the Risk of Bias in non-randomized Studies of Interventions (ROBINS-I) tool was used; The items used for the assessment of each study included bias in the following: due to confounding, in selection of participants into the study, in classification of interventions, due to deviations from the intended interventions, due to missing outcome data, in measurement of the outcome and in the selection of the reported results. For studies with no control group, the NIH Quality Assessment Tool for Before-After (Pre-Post) Studies with No Control Group was used; studies were rated as good, fair or poor quality. Disagreements were resolved by consensus.

### Quality of evidence assessment

Two independent reviewers, working in duplicate, rated the certainty of evidence from included studies using the GRADE approach [17]. Quality of evidence was assessed for: (1) Hospital admission rates, (2) Surgery, (3) Drop-out rate from the adult clinic and transition program, (4) Drug toxicity, (5) Clinical attendance rates. Domains evaluated were the risk of bias of included studies for each particular outcome, the inconsistency of results, indirectness of evidence, imprecision of results, risk of publication bias, and effect size. The estimates of effect for each outcome were graded as high, moderate, low, and very low certainty. As in previous phases, all disagreements were resolved by consensus.

### Data synthesis and statistical analysis

A narrative synthesis of the studies that met our inclusion criteria was conducted. When possible, meta-analyses were performed to estimate the transition program effect over the prespecified outcomes in our PROSPERO registry. When multiple groups were available in one study, we split the shared group into the necessary groups to include multiple independent comparisons, following the Cochrane Handbook for Systematic Reviews of Interventions [18].

For meta-analyses, random-effects models were used to estimate outcome measures assuming high heterogeneity between studies and a true effect for each study. When a low-heterogeneity was expected, fixed-effects models were assessed assuming that treatment effects are equal between the included studies. High heterogeneity was defined as a p-value of < 0.10 for the test of heterogeneity across trials and > 50% for the measure of inconsistency (I^2^). When events were evaluated, odds ratios were used to determine the effect size, we used a modified Mantel–Haenszel meta-analysis with Peto’s method. Meta-analysis data synthesis was performed using R (Version 4.0) with R studio (version 1.2.5001) using the packages metafor and meta.

## Results

### Study selection

**Fig. 1:**
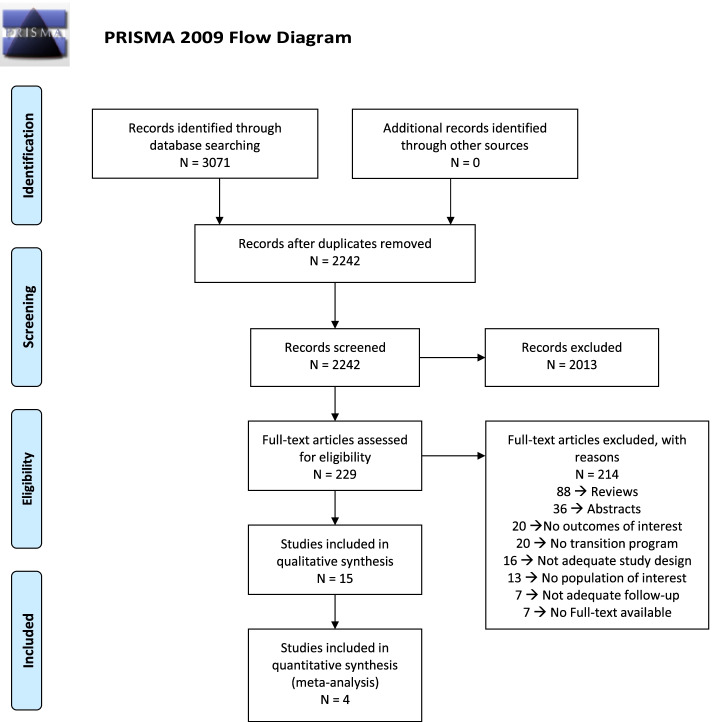
PRISMA Flow-diagram

A total of 2242 records were identified through our search of which 15 studies [9, 19, 20, 21, 22, 23, 24, 25, 26, 27, 28, 29, 30, 31, 32], from 2007 to 2021, met our inclusion criteria. Of these, only one was an RCT (24), 13 were observational studies [9, 19, 20, 21, 22, 23, 26, 27, 28, 29, 30, 31, 32] and one was a complementary study from the one published by McDonough et al. [25] The complete flow-diagram can be found on Fig. 1.

### Study characteristics

A total of 1709 patients were included in our study. The studies’ sample size ranged from 35 to 325 patients. Eight studies included exclusively IBD patients [19, 20, 21, 23, 27, 28, 29, 31], four only juvenile idiopathic arthritis (JIA) [9, 24, 30, 33], one more than one jRMD [32], and two a wide spectrum of jCID [22, 26]. The studies main characteristics are presented in Table 1. Of the three studies that reported remission rates at baseline [20, 21, 29], a total of 184/326(56.4%) patients were in remission at transfer and 114/471 (24.2%) presented active disease (data reported from six studies [19, 20, 21, 29, 30, 31]), however, this information was only available from IBD patients.

**Table 1:** Characteristics of the included studies

Study	Country	Study type	Subjects included	Transition / No transition	Transition description	Setting	Diseases included	Age at transfer (years)	Transition staff	Program duration (months)	Follow-up (months)
Cole, 2015[29]	UK	Retrospective cohort	128	44 / 28	Joint consultations between adult and pediatric gastroenterologists starting at 15 years old	Inflammatory Bowel Disease unit	IBD	16 or older	Pediatric and adult physician, nurse, others	NR	38 (12–47)
Jensen, 2015[30]	USA	Prospective Cohort	236	219 / 26	Assessment for transition awareness and readiness to start the process with social worker (coordinator). Specific workbooks were provided and established written transition goals that were followed and discussed between patients, parents and the transition coordinator. The transfer was done when the pediatric rheumatologist seemed appropriate	Pediatric Rheumatology Clinic	JIA	16 or older	Social worker	Not standardized	6–8
Van den Brink, 2019[19]	Netherlands	Prospective cohort	35	35 / 0	Two multidisciplinary teams (pediatric and adult) discussed all patients before starting. At least four visits per year with pediatric team and once a year with adult team. Transition was made at 18 years old	NR	IBD	18	Pediatric and adult physician, nurse, others	13 (5–18)	12
Otto, 2019[20]	Hungary	Retrospective cohort	45	21 / 24	Joint sessions between pediatric and adult experts every six months to evaluate families of 16 years old adolescents that would transfer at 18 years old	Pediatric Gastroenterology Outpatient Clinic	IBD	NR	Pediatric and adult physician, nurse	NR	NR
Sattoe, 2020[31]	Netherlands	Cohort	110	56 / 54	A multidisciplinary team visited every three months patients aged 16 to 18 for three appointments, a fourth appointment was made with the adult care professional	Adult Gastroenterology Department	IBD	11–17	Pediatric and adult physician, nurse	12	24–48
Shaw, 2006[25]	UK	Prospective cohort	308	308 / 0	Individualized Transition Plans (ITP) were created for young persons and their parents in terms of transition, health, home and school. Three steps were evaluated as early (11–13 years), middle (14–16 years) and late (17 years and over) adolescents. Every ITP was self-completed and reviewed at the clinic every 6 months	NR	JIA	NR	Nurse, Physiotherapist, others	22	NR
McDonagh, 2007[9]											
Hilderson, 2015[24]	Belgium	Prospective cohort	46	23 / 23	Five-step program that started with two appointments with the transition coordinator that provided information and support to the patient and was available by telephone, a information day for adolescents and parents, an individualized transfer plan and the final transfer	Pediatric Rheumatology Department	JIA	14–16	Social worker	16	NR
Walter, 2018[32]	Netherlands	Prospective cohort	154	78 / 76	ITP program started early at 12–14 years, the time of transfer is decided by the patient and physicians at 17–18 years old	Pediatric Rheumatology Department	JIA, SLE, others	NR	Pediatric and adult physician, nurse	NR	36
Cramm, 2013[26]	Netherlands	Retrospective cohort	115	31 / 69	Multicentered effort. Every center used a combination of interventions: information leaflets and websites, checklist for transition, patient reported outcomes (QoL instruments), transition coordinator, transition clinic, structural consultations, group sessions	NR	Type I DM, JIA, NMD	12–25	Pediatric and adult staff	12	12
Testa, 2018[27]	Italy	Retrospective cohort	45	24 / 21	One or two joint sessions between patient, family, pediatric and adult gastroenterologist	Pediatric and Adult Gastroenterology Department	IBD	NR	Pediatric and adult physicians	NR	12
Corsello, 2021[28]	Italy	Prospective cohort	106	43/ 63	Two joint sessions with pediatric and adult gastroenterologists. The first session was to examine previous medical history and planning the time of transition. The second session was to give the patients the possibility to discuss about future plans and therapies in a more autonomous and conscious way	Pediatric Center	IBD	19	Pediatric and adult physicians	NR	18
Gray, 2019[21]	USA	Retrospective cohort	153	82/ 135	Annual meeting with transition coordinator and families for 15–20 min that was followed by phone calls or e-mails three months later to follow up goals set during the meeting. Meetings started at 14 years old and transition readiness was assessed for transfer	Pediatric IBD Clinic	IBD	14–18	Social worker	NR	NR
Schmidt, 2015[22]	Germany	Quasi experimental study	325	53 / 46	Group training workshops were offered two consecutive days for a minimum group of four adolescents. Consisted of eight modules each of 60–90 min duration	NR	Type 1 DM, CF, IBD	15 or older	Psychologist and pediatrician	2 days	6
Schütz, 2019[23]	Germany	Retrospective cohort	35	11 / 24	Joint consultation at 18-year-old with pediatric and adult gastroenterologist without parents before the first visit at the adult clinic	Pediatrics Department	IBD	NR	Pediatric and adult physicians	NR	24

*CF* cystic fibrosis, *DM* diabetes mellitus, *JIA* juvenile idiopathic arthritis, *NMD* neuromuscular disorders, *SLE* Systemic Lupus Erythematosus

### Transition program

The age before transfer was reported only on six studies [20, 21, 31], with a mean age of 17 years (± 3). Duration of disease before transfer ranged from 3 to 8 years.

The mean TP duration was 12.5 months (± 7.2), a mean post-transfer follow-up of 20.9 months (± 16.6), with a mean of 2.3 visits (± 0.9). The TP descriptions across the studies are described in Table 1.

### Disease related outcomes

**Fig. 2:**
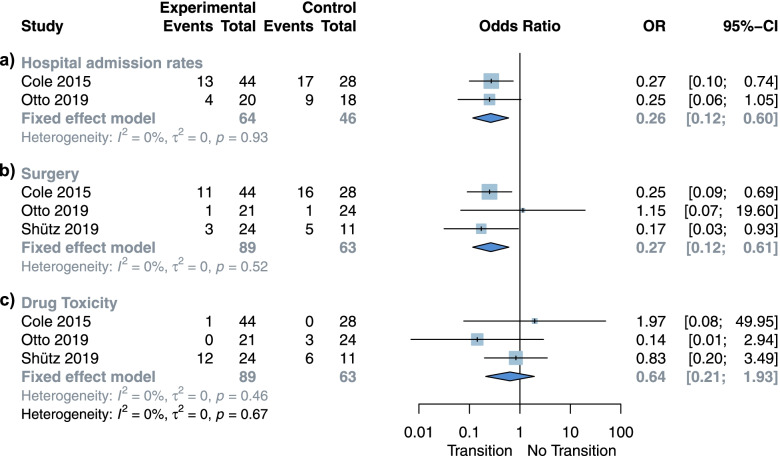
Meta-analysis for disease related outcomes. **a** Hospital admission rates. **b** Surgeries performed. **c** Drug toxicity

Results from our meta-analysis showed reduction on hospital admission rates for those with TP (OR 0.28; 95% CI 0.13 to 0.61; I 2 = 0%; *p* = 0.97 Fig. 2a) this outcome was evaluated during the first two years after transfer. This finding was obtained from two studies [20, 29], comprising the data of 110 IBD patients (64 on TP, 46 with control). Similarly, the pooled analysis of three studies [20, 23, 29], with a total sample size of 152 IBD patients (89 on TP, 63 control group) showed a reduction in the rates of surgeries performed on the group that received a TP (OR 0.26; 95% CI 0.12 to 0.59; I 2 = 0%; *p* = 0.50 Fig. 2b). Regarding drug toxicity, no difference was seen between the two groups (OR 0.61; 95% CI 0.13 to 2.83; I 2 = 29%; *p* = 0.25 Fig. 2c). Disease activity could not be analyzed because of the low number of studies reporting this data.

The overall certainty *per* GRADE approach for this estimate is low to very low. (Supplementary Table 3).

### Program adherence

**Fig. 3:**
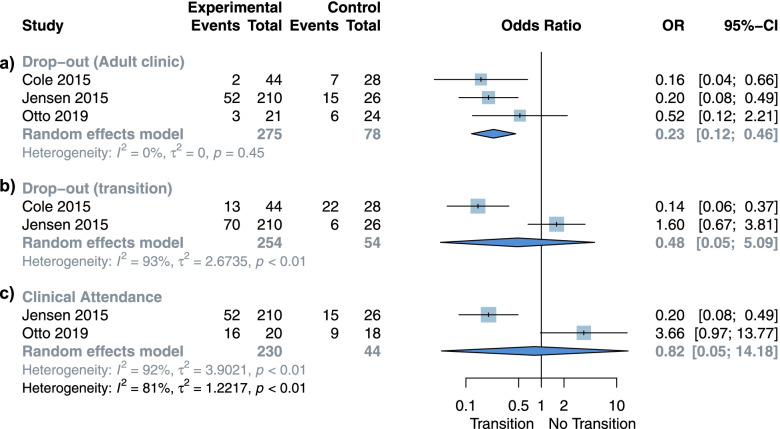
Meta-analysis for program adherence outcomes. **a** Adult clinic drop-out rates. **b** Transition clinic drop-out rates. **c** Overall clinical attend

Three studies reported drop-out rates after transition (adult clinic) (20, 29, 30), comprising 353 IBD and jRMD patients (275 on TP, 78 control group). Our analysis showed lower drop-out rates from the adult clinic when patients were provided with a TP (OR 0.23; 95% CI 0.12 to 0.46; I 2 = 0%; *p* = 0.88 Fig. 3a); however, no effect was seen on the drop-out rates during the transition period (OR 0.48; 95% CI 0.05 to 5.09; I 2 = 93%; *p* < 0.01 Fig. 3b). Clinical attendance did not show a statistically significant difference between the two groups in our analysis (OR 0.82; 95% CI 0.05 to 14.18; I 2 = 92%; *p* < 0.01 Fig. 3c).

The overall certainty *per* GRADE approach for this estimate is low to very low.

### Satisfaction

A total of seven studies reported satisfaction [9, 19, 22, 26, 30, 31, 32]. Two studies used “Mind the Gap” questionnaire [9, 26], one “On your own feet transfer-VAS” [32],one satisfaction with health care (CHS-SUN self) [22] and the other four were self-reported satisfaction questionnaires [9, 19, 30, 31]. More detailed information about the scales used on satisfaction and other outcomes can be found on Table 2.

**Table 2:** Transition program outcomes

Study	Active disease			Satisfaction		HRQoL		
	At transfer	At follow-up	Scale ^a^	At follow-up	Scale ^a^	At transfer	At follow-up	Scale ^a^
**Cole, 2015** [29]	Tr: 14/44 (31.8%) nTr: 17/28 (77.3%)	NR	NR	NR	NR	NR	NR	NR
**Jensen, 2015** [30]	NR	NR	NR	Tr: 42 (range 16—50) nTr: NR	10-item questionnaire created by authors. Range between 5 (negative) and 50 (positive)	NR	NR	NR
**Van den Brink, 2019** [19]	Tr: 5/35 (14.3%) nTr: NA	NR	NR	Tr: < 5.5 0 (0%) 5.5–7 11 (31.4%) > 7 24 (68.6%) nTr: NA	Graded by the patients on a 10-point scale	NR	NR	NR
**Otto, 2019** [20]	Tr: 4/21 (19%) nTr: 5/24 (21%)	Tr: 1/21 4.8% nTr: 6/24 25%	NR	NR	NR	NR	NR	NR
**Sattoe, 2020** [31]	Tr: 13/56 (23.6%) nTr: 27/54 (50%)	Tr: 2/30 (6.9%) nTr: 6/24 25%	NR	Tr: 7.4 (SD 1.3) nTr: 6.8 (SD 1.2)	Graded by the patients on a 10-point scale	NR	Tr: 78.3 (SD 23.8) nTr: 77.6 (SD 15.4)	Paediatric Quality of Life Inventory Young Adult. Range between 0 and 100 (highest HRQoL)
**Shaw, 2006** **McDonagh, 2007** [9, 33]	NR	NR	PGA, number of active and limited joints, VAS-global, VAS-pain	Tr: -0.3 (-3.5, 6.0) nTr: NA	Mind the Gap. Range between − 7 (most satisfied) and 7 (most unsatisfied)	Tr: 2.7 (IQR 1,6.8) nTr: NA	Tr: -0.3 (IQR -3.8, 5.6) nTr: NA	Juvenile Arthritis Quality of Life Questionnaire (JAQQ). Range between 1 to 7 (lower HRQoL)
**Hilderson, 2015** [24]	Tr: 10/23 (43.5%) nTr: 9/23 (39%)	NR	CHAD-QI	NR	NR	NR	NR	NR
**Walter, 2018** [32]	NR	NR	Number of active and painful joints, ESR	Tr: 7.5 (SD 1.9) / 74.5 (SD 12.1) nTr: 7.7 (SD 0.8) / 72 (SD 14.7)	Visual analogue scale (VAS) On your own feet (OYOF-TES)	NR	NR	NR
**Cramm, 2013** [26]	NR	NR	NR	Tr: 0.3 (0.9) nTr: NA	Mind the Gap. Range between − 7 (most satisfied) and 7 (most unsatisfied)	NR	Tr: Emotional 74.8 (SD 17.6) Physical 61.8 (SD 16.9) Social 76 (SD 16.6) nTr: NA	DISABKIDS condition-generic module questionnaire
**Testa, 2018** [27]	NR	NR	NR	NR	NR	NR	NR	NR
**Corsello, 2021** [28]	Tr: 20/82 (24.4%) nTr: NA	NR	NR	NR	NR	NR	Tr: 53.9 (SD 9.8) nTr: NA	SIBDQ
**Gray, 2019** [21]	Tr: 42/135 (31.1%) nTr: 4/18 (23.5%)	Tr: 24/135 (17.8%) nTr: 2/18 (11.7%)	PGA	NR	NR	NR	NR	NR
**Schmidt, 2015** [22]	NR	NR	NR	Tr: 4.04 (SD 0.92) nTr: NR	CHS-SUN self	NR	NR	EUROHIS QOL-8 WHOQOL-Bref DISABKIDS Chronic Generic Measure
**Schütz, 2019** [23]	NR	NR	NR	NR	NR	NR	Tr: 170 (SD 27) nTr: 158 (SD 39)	IBDQ

*Tr* Transition intervention group, *nTr* Control group (standard of care, non-transitional care, or transfer to adult clinic), *NR* Not reported, *NA* Not available (i.e. study with no control group), *a Scale* instrument, tool or definition used for the assessment of the outcome

^a^Scale, instrument, tool or definition used for the assessment of the outcome

### Health related quality of life

Seven studies reported HRQoL using a scale [9, 22, 23, 25, 26, 28, 31], one used the Juvenile Arthritis Quality of Life Questionnaire (JAQQ) [9], one used the Short Inflammatory Bowel Disease Questionnaire (SIBDQ) [28],one the Inflammatory Bowel Disease Questionnaire (IBDQ) [23], two used the DISABKIDS condition-generic module questionnaire [22, 26] and one used the Paediatric Quality of Life Inventory [31].

### Related knowledge

Only three studies reported disease related knowledge [19, 25, 32]. One used the IBD-yourself questionnaire [19] and the other used a 16-item measure designed by the authors [9] one “On your own feet transfer-TES” [32].

### Risk of bias

A total of seven studies were appraised using the ROBINS-I tool, of which six were deemed at serious risk of bias [20, 23, 24, 29, 31, 32] and one at critical risk of bias [21] due to confounding bias. In contrast, five studies were appraised using the NIH Before-After tool, of which three studies were deemed at fair quality [9, 27, 33] and two considered of poor quality [26, 28]. Finally, only one study was evaluated using the Cochrane’s RoB 2 [34], and when assessing for the primary outcome the overall risk of bias was appraised as high.

### GRADE assessment

The global assessment of quality of evidence exhibited low certainty in the estimates of effect in surgery, hospital admission rates, adult drop-out rates, but very low confidence on transition drop-out rates, drug toxicity and clinical attendance.

## Discussion

Our systematic review showed that a TP could lower drop-out rates when transferred to an adult clinic in patients with IBD and jRMD. Also, hospital admission rates and total of surgeries performed decreased in patients with IBD. No statistically significant difference was observed regarding drug toxicity, but due to the low number of studies included and the very low confidence in this result, we cannot make a definitive conclusion on this outcome. We found a rather diverse reporting of satisfaction, quality of life and disease related knowledge, therefore, do a meta-analysis on that information was not appropriate. Even though satisfaction was the most common outcome reported, the use of non-validated self-made scales made the analysis and comparison of these outcomes challenging.

Previous reviews have evaluated transitional care programs on a myriad of chronic illnesses; Crowley et al. [35] provided a narrative systematic review evaluating the effectiveness of transitional care programs in young people with diabetes mellitus, JIA and cystic fibrosis. They only found evidence regarding diabetes mellitus and, similarly to our review, one of the only statistically significant results were seen on clinical attendance rates. Five years later, Clemente et al. [36] critically appraised information available on TP in jRMDs, finding a high variability in processes and outcomes, thus the need for standardization on reporting was concluded. They also emphasize the need of a written transition policy, early entry into transition and the assurance of a competent transition coordinator. On the same field, a review by McDonagh and Farre [37] insight on the lack of a gold standard outcome measure for transition and found two studies reporting improvement on follow-up when a TP was established. In contrast, Rohatinsky et al. [38] performed a scoping review of healthcare transition in patients with IBD. They found that although the articles published on this topic have increased over the last 9 years, a lack of valid and reliable instruments to assess transition readiness was notorious. Our study synthetized relevant evidence focused on the outcomes of the TP on jCID, adding a meta-analysis that shown an overall positive effect of those interventions.

The remarking recent interest in the transition process is highlighted by the recommendations proposed by international organizations, although with some significant differences. The European League Against Rheumatism (EULAR) and Paediatric Rheumatology European Society (PReS) taskforce standards for transitional care in young people with jRMDs, considered the ideal start of transition at 11 years and essential by 14;[39] Similarly, the National Institute for Health and Care Excellence (NICE) states that transition planning should start at 13 or 14 years.[40] In contrast, the North American Society for Pediatric Gastroenterology, Hepatology and Nutrition (NASPGHAN) is more lenient on its recommendations for patients with IBD, advising transition on patients up to 18 years of age [41]. Similarly, the American Academy of Pediatrics (AAP), with the endorsement of the American Academy of Family Physicians (AAFP) and the American College of Physicians (ACP), provide a timeline introducing core elements into pediatric practices and mentions the transfer to adult-centered care from ages 18 to 21 [5]. In our review, the mean age of transfer of the studies included was 17 years, a considerable difference of 6 years after the ideal proposed age by EULAR/PReS. All the organizations agree that limited quality indicators exist to adequately assess whether a transition was successful or not.

A contributing factor that could explain the lack in proper standardization of TP could be explained by the diverse health policies implemented in each country and the probable lack of funding. Hepburn et al. [42] discussed how governmental programs are usually universally applied to a population, making funding difficult. This issue, added to the limited cost-effectiveness analysis of these programs, makes its financing even more difficult, and therefore, their implementation.

Certain limitations in our study need to be acknowledged. The heterogeneity of the TP and the definition of the outcomes such as clinical attendance and drop-out rates, the lack of a control group in several studies, the myriad of scales used to assess different outcomes, make it inadequate to perform direct comparisons between groups and limit the analyses performed. It is important to notice that the information available are only from a limited number of jCID. Relevant conditions such as juvenile psoriasis, autoinflammatory syndromes and most of the jRMDs were excluded from studies. Thus, our conclusions should primarily be applied to patients with IBD and JIA.

Despite this, a strength in our study is the extensive literature search performed, assuring the inclusion of most of the jCID, which gives our review the capacity to provide a definitive conclusion on what evidence is missing and what are TPs lacking. Another strength of our review is the use of GRADE, providing an adequate assessment that can be translated immediately into clinical practice, considering the quality of the evidence and the strength of the results presented.

### Implications for future research

This work shows the benefits of TP on young people with jCID, but confirms the need for standardized measures. Each program should report the same outcomes to be able to perform future cost-effective analyses, endorse funding and promote generalizability on these programs. Additionally, exploring different alternatives for TPs could be a relevant research area, this includes low-cots intervention and taking advantage of technology, adapting to new modalities, such as online programs. Finally, it is important to generate information on TP for other jCID, an underreported topic up to now.

## Conclusion

Our study found that TPs could be a determining factor in preventing hospital admission rates, surgeries needed and adult clinical attendance rates. However, this study highlights the need of stronger, guideline compliant TPs in not only IBD and jRMD, but on every jCID, to improve the care of young people with these conditions. Additionally, further research is needed to measure the success of the process to determine the best transitional model.

## Supplementary Information

**Additional file 1:** **Supplementary Table 1.** Search strategy- March 16, 2021. **Supplementary Table 2.** PRISMA Checklist. **Supplementary Table 3.** GRADE Assessment.

## Data Availability

The datasets used and/or analyzed during the current study are available from the corresponding author on reasonable request.

## Abbreviations

jCID: Juvenile chronic inflammatory systemic diseases
IBD: Inflammatory bowel disease
jRMD: Juvenile-onset rheumatic and musculoskeletal diseases
TP: Transitional care program
JIA: Juvenile idiopathic arthritis
JAQQ: Juvenile Arthritis Quality of Life Questionnaire
SIBDQ: Short Inflammatory Bowel Disease Questionnaire
IBDQ: Inflammatory Bowel Disease Questionnaire
