# Supplementary material for: Outcomes of transitional care programs on adolescent chronic inflammatory systemic diseases: systematic review and meta-analyses
Source: Pediatr Rheumatol Online J. 2022 Feb 17;20:15. doi: 10.1186/s12969-022-00670-1 (PMC8851760; doi:10.1186/s12969-022-00670-1)
Supplement: Supplementary file 1 — Additional file 1: Supplementary Table 1. Search strategy- March 16, 2021. Supplementary Table 2. PRISMA Checklist. Supplementary Table 3. GRADE Assessment. [file 12969_2022_670_MOESM1_ESM.pdf]

SUPPLEMENTARY MATERIAL

Supplementary Table 1. Search strategy- March 16, 2021

Database	Search Term	Search Results Before deduplication	Search Results After deduplication
Scopus	(TITLE-ABS-KEY ("Arthritis, Juvenile Chronic" OR "Arthritis, Juvenile Enthesitis-Related" OR "Arthritis, Juvenile Idiopathic" OR "Arthritis, Juvenile PsORiatic" OR "Arthritis, Juvenile Rheumatoid" OR "Arthritis, Juvenile Systemic" OR "Chronic Arthritis, Juvenile" OR "Enthesitis Related Arthritis, Juvenile" OR "Enthesitis-Related Arthritis, Juvenile" OR "Idiopathic Arthritis, Juvenile" OR "Juvenile Arthritis" OR "Juvenile Chronic Arthritis" OR "Juvenile Enthesitis-Related Arthritis" OR "Juvenile Idiopathic Arthritis" OR "Juvenile Oligoarthritis" OR "Juvenile Onset Still Disease" OR "Juvenile Onset Stills Disease" OR "Juvenile PsORiatic Arthritis" OR "Juvenile Rheumatoid Arthritis" OR "Juvenile Systemic Arthritis" OR "Juvenile-Onset Still Disease" OR "Juvenile-Onset Still's Disease" OR "Juvenile-Onset Stills Disease" OR "Oligoarthritis, Juvenile" OR "Polyarthritis, Juvenile, Rheumatoid FactOR Negative" OR "Polyarthritis, Juvenile, Rheumatoid FactOR Positive" OR "PsORiatic Arthritis, Juvenile" OR "Rheumatoid Arthritis, Juvenile" OR "Still Disease, Juvenile Onset" OR "Still Disease, Juvenile-Onset" OR "Still's Disease, Juvenile Onset" OR "Still's Disease, Juvenile-Onset" OR "Stills Disease, Juvenile-Onset" OR "Systemic Arthritis, Juvenile" OR "Colitis, Ulcerative" OR "Colitis Gravis" OR "Idiopathic Proctocolitis" OR "InflammatORy Bowel Disease, Ulcerative Colitis Type" OR "Ulcerative Colitis" OR "InflammatORy Bowel Diseases" OR "Lupus Erythematosus, Systemic" OR "Libman-Sacks Disease" OR "Lupus Erythematosus Disseminatus" OR "Systemic Lupus Erythematosus" OR "Dermatomyositis" OR "Childhood Type Dermatomyositis" OR "Dermatomyositis, Childhood Type" OR "Dermatomyositis, Juvenile" OR "Dermatopolymyositis" OR "Juvenile Dermatomyositis" OR "Juvenile Myositis" OR "Myositis, Juvenile" OR "Polymyositis Dermatomyositis" OR "Polymyositis-Dermatomyositis" OR "Sclerosis, Systemic" OR "Systemic Scleroderma" OR "Systemic Sclerosis" OR "Scleroderma, Systemic" OR "Diffuse Cutaneous Systemic Sclerosis" OR "Diffuse Scleroderma" OR "Diffuse Systemic Sclerosis" OR "Scleroderma, Progressive" OR "Sclerosis, Progressive Systemic" OR "Sudden Onset Scleroderma" OR "Scleroderma, Diffuse" OR "Arthritis, PsORiatic" OR "PsORiasis Arthropathica" OR "PsORiasis, Arthritic" OR "PsORiatic Arthritis" OR "PsORiatic Arthropathy" OR "PsORiasis" OR "Palmoplantaris Pustulosis" OR "Pustular PsORiasis of Palms and Soles" OR "Pustulosis of Palms and Soles" OR "Pustulosis Palmaris et Plantaris" OR "Rheumatic diseases" OR "Rheumatic conditions" OR "Churg-Strauss Syndrome" OR "Allergic Angiitis" OR "Allergic Angiitis and Granulomatosis" OR "Allergic Granulomatosis" OR "Allergic Granulomatous and Angiitis" OR "Allergic Granulomatous Angiitis" OR "Angiitis, Allergic Granulomatous" OR "Eosinophilic Granulomatous Vasculitis" OR "Granulomatous Allergic Angiitis" OR "Vasculitis, Churg-Strauss" OR "Purpura, Schoenlein-Henoch" OR "Allergic Purpura" OR "Anaphylactoid Purpura" OR "Henoch Purpura" OR "Henoch Schonlein Purpura" OR "Purpura, Schoenlein-Henoch" OR "Purpura, Rheumatoid" OR "Henoch-Schoenlein Purpura" OR "Henoch-Schonlein Purpura" OR "Purpura HemORrhagica" OR "Purpura, Nonthrombocytopenic" OR "Purpura, Nonthrombopenic" OR "Purpura, Schonlein-Henoch" OR "Rheumatoid Purpura" OR "Schoenlein-Henoch Purpura" OR "Vasculitis, HemORrhagic" OR "Rheumatoid Vasculitis" OR "Systemic Vasculitis" OR "Vasculitis" OR "ANCA-Associated Vasculitides" OR "ANCA-Associated Vasculitis" OR "Pauci-Immune Vasculitis" OR "Anti-Neutrophil Cytoplasmic Antibody-Associated Vasculitis" OR "Mucocutaneous Lymph Node Syndrome" OR "Kawasaki Disease" OR "Kawasaki Syndrome" OR "Lymph Node Syndrome, Mucocutaneous" OR "Ankylosing Spondylarthritis" OR "Ankylosing Spondylitis" OR "Ankylosing Spondyloarthritis" OR "Bechterew Disease" OR "Bechterew's Disease" OR "Marie-Struempell Disease" OR "Rheumatoid Spondylitis" OR "Spondylarthritis Ankylopoietica" OR "Spondylitis Ankylopoietica" OR "Spondyloarthritis Ankylopoietica" OR "Spondylitis, Ankylosing" OR "autoinflammatORy syndrome" OR "Antiphospholipid Syndrome" OR "Anti Phospholipid Antibody Syndrome" OR "Anti Phospholipid Syndrome" OR "Anti-Phospholipid Antibody Syndrome" OR "Anti-Phospholipid Syndrome" OR "Antibody Syndrome, Anti-Phospholipid" OR "Antibody Syndrome, Antiphospholipid" OR "Antiphospholipid Antibody Syndrome" OR "Antiphospholipid Antibody Syndromes" OR "Hughes Syndrome" OR "Syndrome, Anti-Phospholipid" OR "Syndrome, Anti-Phospholipid Antibody" OR "Syndrome, Antiphospholipid" OR "Syndrome, Antiphospholipid Antibody" OR "Syndrome, Hughes" OR "Behcet Syndrome" OR "Adamantiades Behcet Disease" OR "Adamantiades-Behcet Disease" OR "Adamantiades-Behcet Diseases" OR "Behcet Disease" OR "Behcet Triple Symptom Complex" OR "Behcet's Syndrome" OR "Behcets Syndrome" OR "Behçet Disease" OR "Behçet Diseases" OR "Complex, Triple Symptom" OR "Complices, Triple Symptom" OR "Disease, Adamantiades-Behcet" OR "Disease, Behçet" OR "Diseases, Adamantiades-Behcet" OR "Diseases, Behçet" OR "Old Silk Route Disease" OR "Symptom Complex, Triple" OR "Symptom Complices, Triple" OR "Triple Symptom Complex" OR "Triple Symptom Complices" OR "Triple-Symptom Complex" OR "Sjogren's Syndrome" OR "Sicca Syndrome" OR "Sjogren Syndrome")) AND (("transitional care program" OR "transition to adult care" OR "transition pathway" OR "transition program" OR "transition programme" OR "brief transition programme" OR "health program" OR "patient care planning " OR "Adolescent transition to adult care"))	1534	1113
Medline	("Arthritis, Juvenile Chronic" or "Arthritis, Juvenile Enthesitis-Related" or "Arthritis, Juvenile Idiopathic" or "Arthritis, Juvenile PsORiatic" or "Arthritis, Juvenile Rheumatoid" or "Arthritis, Juvenile Systemic" or "Chronic Arthritis, Juvenile" or "Enthesitis Related Arthritis, Juvenile" or "Enthesitis-Related Arthritis, Juvenile" or "Idiopathic Arthritis, Juvenile" or "Juvenile Arthritis" or "Juvenile Chronic Arthritis" or "Juvenile Enthesitis-Related Arthritis" or "Juvenile Idiopathic Arthritis" or "Juvenile Oligoarthritis" or "Juvenile Onset Still Disease" or "Juvenile Onset Stills Disease" or "Juvenile PsORiatic Arthritis" or "Juvenile Rheumatoid Arthritis" or "Juvenile Systemic Arthritis" or "Juvenile-Onset Still Disease" or "Juvenile-Onset Still's Disease" or "Juvenile-Onset Stills Disease" or "Oligoarthritis, Juvenile" or "Polyarthritis, Juvenile, Rheumatoid FactOR Negative" or "Polyarthritis, Juvenile, Rheumatoid FactOR Positive" or "PsORiatic Arthritis, Juvenile" or "Rheumatoid Arthritis, Juvenile" or "Still Disease, Juvenile Onset" or "Still Disease, Juvenile-Onset" or "Still's Disease, Juvenile Onset" or "Still's Disease, Juvenile-Onset" or "Stills Disease, Juvenile-Onset" or	241	125

	"Systemic Arthritis, Juvenile" or "Colitis, Ulcerative" or "Colitis Gravis" or "Idiopathic Proctocolitis" or "InflammatORy Bowel Disease, Ulcerative Colitis Type" or "Ulcerative Colitis" or "InflammatORy Bowel Diseases" or "Lupus Erythematosus, Systemic" or "Libman-Sacks Disease" or "Lupus Erythematosus Disseminatus" or "Systemic Lupus Erythematosus" or "Dermatomyositis" or "Childhood Type Dermatomyositis" or "Dermatomyositis, Childhood Type" or "Dermatomyositis, Juvenile" or "Dermatopolymyositis" or "Juvenile Dermatomyositis" or "Juvenile Myositis" or "Myositis, Juvenile" or "Polymyositis Dermatomyositis" or "Polymyositis-Dermatomyositis" or "Sclerosis, Systemic" or "Systemic Scleroderma" or "Systemic Sclerosis" or "Scleroderma, Systemic" or "Diffuse Cutaneous Systemic Sclerosis" or "Diffuse Scleroderma" or "Diffuse Systemic Sclerosis" or "Scleroderma, Progressive" or "Sclerosis, Progressive Systemic" or "Sudden Onset Scleroderma" or "Scleroderma, Diffuse" or "Arthritis, PsORiatic" or "PsORiasis Arthropathica" or "PsORiasis, Arthritic" or "PsORiatic Arthritis" or "PsORiatic Arthropathy" or "PsORiasis" or "Palmoplantaris Pustulosis" or "Pustular PsORiasis of Palms and Soles" or "Pustulosis of Palms and Soles" or "Pustulosis Palmaris et Plantaris" or "Rheumatic diseases" or "Rheumatic conditions" or "Churg-Strauss Syndrome" or "Allergic Angiitis" or "Allergic Angiitis and Granulomatosis" or "Allergic Granulomatosis" or "Allergic Granulomatous and Angiitis" or "Allergic Granulomatous Angiitis" or "Angiitis, Allergic Granulomatous" or "Eosinophilic Granulomatous Vasculitis" or "Granulomatous Allergic Angiitis" or "Vasculitis, Churg-Strauss" or "Purpura, Schoenlein-Henoch" or "Allergic Purpura" or "Anaphylactoid Purpura" or "Henoch Purpura" or "Henoch Schonlein Purpura" or "Purpura, Schoenlein-Henoch" or "Purpura, Rheumatoid" or "Henoch-Schoenlein Purpura" or "Henoch-Schonlein Purpura" or "Purpura HemORrhagica" or "Purpura, Nonthrombocytopenic" or "Purpura, Nonthrombopenic" or "Purpura, Schonlein-Henoch" or "Rheumatoid Purpura" or "Schoenlein-Henoch Purpura" or "Vasculitis, HemORrhagic" or "Rheumatoid Vasculitis" or "Systemic Vasculitis" or "Vasculitis" or "ANCA-Associated Vasculitides" or "ANCA-Associated Vasculitis" or "Pauci-Immune Vasculitis" or "Anti-Neutrophil Cytoplasmic Antibody-Associated Vasculitis" or "Mucocutaneous Lymph Node Syndrome" or "Kawasaki Disease" or "Kawasaki Syndrome" or "Lymph Node Syndrome, Mucocutaneous" or "Ankylosing Spondylarthritis" or "Ankylosing Spondylitis" or "Ankylosing Spondyloarthritis" or "Bechterew Disease" or "Bechterew's Disease" or "Marie-Struempell Disease" or "Rheumatoid Spondylitis" or "Spondylarthritis Ankylopoietica" or "Spondylitis Ankylopoietica" or "Spondyloarthritis Ankylopoietica" or "Spondylitis, Ankylosing" or "autoinflammatORy syndrome" or "Antiphospholipid Syndrome" or "Anti Phospholipid Antibody Syndrome" or "Anti Phospholipid Syndrome" or "Anti-Phospholipid Antibody Syndrome" or "Anti-Phospholipid Syndrome" or "Antibody Syndrome, Anti-Phospholipid" or "Antibody Syndrome, Antiphospholipid" or "Antiphospholipid Antibody Syndrome" or "Antiphospholipid Antibody Syndromes" or "Hughes Syndrome" or "Syndrome, Anti-Phospholipid" or "Syndrome, Anti-Phospholipid Antibody" or "Syndrome, Antiphospholipid" or "Syndrome, Antiphospholipid Antibody" or "Syndrome, Hughes" or "Behcet Syndrome" or "Adamantiades Behcet Disease" or "Adamantiades-Behcet Disease" or "Adamantiades-Behcet Diseases" or "Behcet Disease" or "Behcet Triple Symptom Complex" or "Behcet's Syndrome" or "Behcets Syndrome" or "Behçet Disease" or "Behçet Diseases" or "Complex, Triple Symptom" or "Complices, Triple Symptom" or "Disease, Adamantiades-Behcet" or "Disease, Behçet" or "Diseases, Adamantiades-Behcet" or "Diseases, Behçet" or "Old Silk Route Disease" or "Symptom Complex, Triple" or "Symptom Complices, Triple" or "Triple Symptom Complex" or "Triple Symptom Complices" or "Triple-Symptom Complex" or "Sjogren's Syndrome" or "Sicca Syndrome" or "Sjogren Syndrome").ab,kw,ti.<br><br>("transitional care program" or "transition to adult care" or "transition pathway" or "transition program" or "transition programme" or "brief transition programme" or "health program" or "patient care planning " or "Adolescent transition to adult care").mp. [mp=ti, ab, hw, tn, ot, dm, mf, dv, kw, fx, dq, nm, kf, ox, px, rx, an, ui, sy]		
Web of Sciende	TS= ("Arthritis, Juvenile Chronic" OR "Arthritis, Juvenile Enthesitis-Related" OR "Arthritis, Juvenile Idiopathic" OR "Arthritis, Juvenile PsORiatic" OR "Arthritis, Juvenile Rheumatoid" OR "Arthritis, Juvenile Systemic" OR "Chronic Arthritis, Juvenile" OR "Enthesitis Related Arthritis, Juvenile" OR "Enthesitis-Related Arthritis, Juvenile" OR "Idiopathic Arthritis, Juvenile" OR "Juvenile Arthritis" OR "Juvenile Chronic Arthritis" OR "Juvenile Enthesitis-Related Arthritis" OR "Juvenile Idiopathic Arthritis" OR "Juvenile Oligoarthritis" OR "Juvenile Onset Still Disease" OR "Juvenile Onset Stills Disease" OR "Juvenile PsORiatic Arthritis" OR "Juvenile Rheumatoid Arthritis" OR "Juvenile Systemic Arthritis" OR "Juvenile-Onset Still Disease" OR "Juvenile-Onset Still's Disease" OR "Juvenile-Onset Stills Disease" OR "Oligoarthritis, Juvenile" OR "Polyarthritis, Juvenile, Rheumatoid FactOR Negative" OR "Polyarthritis, Juvenile, Rheumatoid FactOR Positive" OR "PsORiatic Arthritis, Juvenile" OR "Rheumatoid Arthritis, Juvenile" OR "Still Disease, Juvenile Onset" OR "Still Disease, Juvenile-Onset" OR "Still's Disease, Juvenile Onset" OR "Still's Disease, Juvenile-Onset" OR "Stills Disease, Juvenile-Onset" OR "Systemic Arthritis, Juvenile" OR "Colitis, Ulcerative" OR "Colitis Gravis" OR "Idiopathic Proctocolitis" OR "InflammatORy Bowel Disease, Ulcerative Colitis Type" OR "Ulcerative Colitis" OR "InflammatORy Bowel Diseases" OR "Lupus Erythematosus, Systemic" OR "Libman-Sacks Disease" OR "Lupus Erythematosus Disseminatus" OR "Systemic Lupus Erythematosus" OR "Dermatomyositis" OR "Childhood Type Dermatomyositis" OR "Dermatomyositis, Childhood Type" OR "Dermatomyositis, Juvenile" OR "Dermatopolymyositis" OR "Juvenile Dermatomyositis" OR "Juvenile Myositis" OR "Myositis, Juvenile" OR "Polymyositis Dermatomyositis" OR "Polymyositis-Dermatomyositis" OR "Sclerosis, Systemic" OR "Systemic Scleroderma" OR "Systemic Sclerosis" OR "Scleroderma, Systemic" OR "Diffuse Cutaneous Systemic Sclerosis" OR "Diffuse Scleroderma" OR "Diffuse Systemic Sclerosis" OR "Scleroderma, Progressive" OR "Sclerosis, Progressive Systemic" OR "Sudden Onset Scleroderma" OR "Scleroderma, Diffuse" OR "Arthritis, PsORiatic" OR "PsORiasis Arthropathica" OR "PsORiasis, Arthritic" OR "PsORiatic Arthritis" OR "PsORiatic Arthropathy" OR "PsORiasis" OR "Palmoplantaris Pustulosis" OR "Pustular PsORiasis of Palms and Soles" OR "Pustulosis of Palms and Soles" OR "Pustulosis Palmaris et Plantaris" OR "Rheumatic diseases" OR "Rheumatic conditions" OR "Churg-Strauss Syndrome" OR "Allergic Angiitis" OR "Allergic Angiitis and Granulomatosis" OR "Allergic Granulomatosis" OR "Allergic Granulomatous and Angiitis" OR "Allergic Granulomatous Angiitis" OR "Angiitis, Allergic Granulomatous" OR "Eosinophilic Granulomatous Vasculitis" OR "Granulomatous Allergic Angiitis" OR "Vasculitis, Churg-Strauss" OR "Purpura, Schoenlein-Henoch" OR "Allergic Purpura" OR "Anaphylactoid Purpura" OR "Henoch Purpura" OR "Henoch Schonlein Purpura" OR "Purpura, Schoenlein-Henoch" OR "Purpura, Rheumatoid" OR "Henoch-Schoenlein Purpura" OR "Henoch-Schonlein Purpura" OR "Purpura HemORrhagica" OR "Purpura, Nonthrombocytopenic" OR "Purpura, Nonthrombopenic" OR "Purpura, Schonlein-Henoch" OR "Rheumatoid Purpura" OR "Schoenlein-Henoch Purpura" OR "Vasculitis, HemORrhagic"	67	8

	OR "Rheumatoid Vasculitis" OR "Systemic Vasculitis" OR "Vasculitis" OR "ANCA-Associated Vasculitides" OR "ANCA-Associated Vasculitis" OR "Pauci-Immune Vasculitis" OR "Anti-Neutrophil Cytoplasmic Antibody-Associated Vasculitis" OR "Mucocutaneous Lymph Node Syndrome" OR "Kawasaki Disease" OR "Kawasaki Syndrome" OR "Lymph Node Syndrome, Mucocutaneous" OR "Ankylosing Spondylarthritis" OR "Ankylosing Spondylitis" OR "Ankylosing Spondyloarthritis" OR "Bechterew Disease" OR "Bechterew's Disease" OR "Marie-Struempell Disease" OR "Rheumatoid Spondylitis" OR "Spondylarthritis Ankylopoietica" OR "Spondylitis Ankylopoietica" OR "Spondyloarthritis Ankylopoietica" OR "Spondylitis, Ankylosing" OR "autoinflammatORy syndrome" OR "Antiphospholipid Syndrome" OR "Anti Phospholipid Antibody Syndrome" OR "Anti Phospholipid Syndrome" OR "Anti-Phospholipid Antibody Syndrome" OR "Anti-Phospholipid Syndrome" OR "Antibody Syndrome, Anti-Phospholipid" OR "Antibody Syndrome, Antiphospholipid" OR "Antiphospholipid Antibody Syndrome" OR "Antiphospholipid Antibody Syndromes" OR "Hughes Syndrome" OR "Syndrome, Anti-Phospholipid" OR "Syndrome, Anti-Phospholipid Antibody" OR "Syndrome, Antiphospholipid" OR "Syndrome, Antiphospholipid Antibody" OR "Syndrome, Hughes" OR "Behcet Syndrome" OR "Adamantiades Behcet Disease" OR "Adamantiades-Behcet Disease" OR "Adamantiades-Behcet Diseases" OR "Behcet Disease" OR "Behcet Triple Symptom Complex" OR "Behcet's Syndrome" OR "Behcets Syndrome" OR "Behçet Disease" OR "Behçet Diseases" OR "Complex, Triple Symptom" OR "Complices, Triple Symptom" OR "Disease, Adamantiades-Behcet" OR "Disease, Behçet" OR "Diseases, Adamantiades-Behcet" OR "Diseases, Behçet" OR "Old Silk Route Disease" OR "Symptom Complex, Triple" OR "Symptom Complices, Triple" OR "Triple Symptom Complex" OR "Triple Symptom Complices" OR "Triple-Symptom Complex" OR "Sjogren's Syndrome" OR "Sicca Syndrome" OR "Sjogren Syndrome")		
Embase	(juvenile rheumatoid arthritis or rheumatoid arthritis or ulcerative colitis or systemic lupus erythematosus or dermatomyositis or systemic sclerosis or psoriatic arthritis or pustulosis palmoplantaris or rheumatic disease or Churg Strauss syndrome or anaphylactoid purpura or rheumatoid vasculitis or mucocutaneous lymph node syndrome or submandibular lymph node or ankylosing spondylitis or ankylosing spondylitis or rheumatic disease or antiphospholipid syndrome or Behcet disease or Sjoegren syndrome or Wegener granulomatosis).ti. or (juvenile rheumatoid arthritis or rheumatoid arthritis or ulcerative colitis or systemic lupus erythematosus or dermatomyositis or systemic sclerosis or psoriatic arthritis or pustulosis palmoplantaris or rheumatic disease or Churg Strauss syndrome or anaphylactoid purpura or rheumatoid vasculitis or mucocutaneous lymph node syndrome or submandibular lymph node or ankylosing spondylitis or ankylosing spondylitis or rheumatic disease or antiphospholipid syndrome or Behcet disease or Sjoegren syndrome or Wegener granulomatosis).ab. or (juvenile rheumatoid arthritis or rheumatoid arthritis or ulcerative colitis or systemic lupus erythematosus or dermatomyositis or systemic sclerosis or psoriatic arthritis or pustulosis palmoplantaris or rheumatic disease or Churg Strauss syndrome or anaphylactoid purpura or rheumatoid vasculitis or mucocutaneous lymph node syndrome or submandibular lymph node or ankylosing spondylitis or ankylosing spondylitis or rheumatic disease or antiphospholipid syndrome or Behcet disease or Sjoegren syndrome or Wegener granulomatosis).kw.	204	161

Supplementary table 2. PRISMA Checklist

Section/topic	#	Checklist item	Reported on page #
**TITLE**			
Title	1	Identify the report as a systematic review, meta-analysis, or both.	1
**ABSTRACT**			
Structured summary	2	Provide a structured summary including, as applicable: background; objectives; data sources; study eligibility criteria, participants, and interventions; study appraisal and synthesis methods; results; limitations; conclusions and implications of key findings; systematic review registration number.	3
**INTRODUCTION**			
Rationale	3	Describe the rationale for the review in the context of what is already known.	4
Objectives	4	Provide an explicit statement of questions being addressed with reference to participants, interventions, comparisons, outcomes, and study design (PICOS).	5
**METHODS**			
Protocol and registration	5	Indicate if a review protocol exists, if and where it can be accessed (e.g., Web address), and, if available, provide registration information including registration number.	5
Eligibility criteria	6	Specify study characteristics (e.g., PICOS, length of follow-up) and report characteristics (e.g., years considered, language, publication status) used as criteria for eligibility, giving rationale.	5
Information sources	7	Describe all information sources (e.g., databases with dates of coverage, contact with study authors to identify additional studies) in the search and date last searched.	5
Search	8	Present full electronic search strategy for at least one database, including any limits used, such that it could be repeated.	Suppl
Study selection	9	State the process for selecting studies (i.e., screening, eligibility, included in systematic review, and, if applicable, included in the meta-analysis).	6
Data collection process	10	Describe method of data extraction from reports (e.g., piloted forms, independently, in duplicate) and any processes for obtaining and confirming data from investigators.	6
Data items	11	List and define all variables for which data were sought (e.g., PICOS, funding sources) and any assumptions and simplifications made.	6
Risk of bias in individual studies	12	Describe methods used for assessing risk of bias of individual studies (including specification of whether this was done at the study or outcome level), and how this information is to be used in any data synthesis.	6
Summary measures	13	State the principal summary measures (e.g., risk ratio, difference in means).	7,8
Synthesis of results	14	Describe the methods of handling data and combining results of studies, if done, including measures of consistency (e.g., I^2) for each meta-analysis.	8

Section/topic	#	Checklist item	Reported on page #
Risk of bias across studies	15	Specify any assessment of risk of bias that may affect the cumulative evidence (e.g., publication bias, selective reporting within studies).	6
Additional analyses	16	Describe methods of additional analyses (e.g., sensitivity or subgroup analyses, meta-regression), if done, indicating which were pre-specified.	8
**RESULTS**			
Study selection	17	Give numbers of studies screened, assessed for eligibility, and included in the review, with reasons for exclusions at each stage, ideally with a flow diagram.	8,figure 1
Study characteristics	18	For each study, present characteristics for which data were extracted (e.g., study size, PICOS, follow-up period) and provide the citations.	8,9, Table 1
Risk of bias within studies	19	Present data on risk of bias of each study and, if available, any outcome level assessment (see item 12).	10,11
Results of individual studies	20	For all outcomes considered (benefits or harms), present, for each study: (a) simple summary data for each intervention group (b) effect estimates and confidence intervals, ideally with a forest plot.	9,10, Figure 2-3
Synthesis of results	21	Present results of each meta-analysis done, including confidence intervals and measures of consistency.	9,19
Risk of bias across studies	22	Present results of any assessment of risk of bias across studies (see Item 15).	10
Additional analysis	23	Give results of additional analyses, if done (e.g., sensitivity or subgroup analyses, meta-regression [see Item 16]).	NA
**DISCUSSION**			
Summary of evidence	24	Summarize the main findings including the strength of evidence for each main outcome; consider their relevance to key groups (e.g., healthcare providers, users, and policy makers).	11
Limitations	25	Discuss limitations at study and outcome level (e.g., risk of bias), and at review-level (e.g., incomplete retrieval of identified research, reporting bias).	13
Conclusions	26	Provide a general interpretation of the results in the context of other evidence, and implications for future research.	14
**FUNDING**			
Funding	27	Describe sources of funding for the systematic review and other support (e.g., supply of data); role of funders for the systematic review.	1

From: Moher D, Liberati A, Tetzlaff J, Altman DG, The PRISMA Group (2009). Preferred Reporting Items for Systematic Reviews and Meta-Analyses: The PRISMA Statement. PLoS Med 6(7): e1000097. doi:10.1371/journal.pmed1000097

Supplementary Table 3. GRADE Assessment

Certainty assessment							№ of patients		Effect		Certainty	Importance
№ of studies	Study design	Risk of bias	Inconsistency	Indirectness	Imprecision	Other considerations	Transition program	No transition program	Relative (95% CI)	Absolute (95% CI)		
**Hospital admission rates**												
2	observational studies	serious [a]	not serious	not serious	very serious [b]	all plausible residual confounding would reduce the demonstrated effect	17/64 (26.6%)	25/46 (54.3%)	**OR 0.28** (0.13 to 0.61)	**293 fewer per 1,000** (from 409 fewer to 123 fewer)	⊕⊕◯◯ LOW	IMPORTANT
**Surgery**												
3	observational studies	serious [a]	serious [c]	not serious	serious [b]	all plausible residual confounding would reduce the demonstrated effect	22/89 (24.7%)	22/63 (34.9%)	**OR 0.26** (0.12 to 0.59)	**227 fewer per 1,000** (from 289 fewer to 109 fewer)	⊕⊕◯◯ LOW	CRITICAL
**Drop-out (Adult clinic)**												
3	observational studies	serious [a]	serious [c]	not serious	serious [b]	all plausible residual confounding would reduce the demonstrated effect	57/275 (20.7%)	28/78 (35.9%)	**OR 0.23** (0.12 to 0.46)	**245 fewer per 1,000** (from 296 fewer to 154 fewer)	⊕⊕◯◯ LOW	IMPORTANT
**Drop-out (transition)**												
2	observational studies	serious [a]	very serious [d]	not serious	serious [b]	all plausible residual confounding would reduce the demonstrated effect	83/254 (32.7%)	28/54 (51.9%)	**OR 0.48** (0.05 to 5.09)	**178 fewer per 1,000** (from 467 fewer to 327 more)	⊕◯◯◯ VERY LOW	IMPORTANT
**Drug Toxicity**												
2	observational studies	serious [a]	very serious [d]	not serious	very serious [b]	all plausible residual confounding would reduce the demonstrated effect	13/89 (14.6%)	9/63 (14.3%)	**OR 0.61** (0.13 to 2.83)	**51 fewer per 1,000** (from 122 fewer to 178 more)	⊕◯◯◯ VERY LOW	CRITICAL
**Clinical Attendance**												
2	observational studies	serious [a]	very serious [d]	not serious	very serious [b]	all plausible residual confounding would reduce the demonstrated effect	68/230 (29.6%)	24/44 (54.5%)	**OR 0.82** (0.05 to 14.18)	**49 fewer per 1,000** (from 489 fewer to 399 more)	⊕◯◯◯ VERY LOW	IMPORTANT

CI: Confidence interval; **OR:** Odds ratio
a. a. Due to major issues regarding confounding bias
b. Optimal information size is not met
c. Point estimates vary across studies with wide condifence intervals.
d. Point estimates vary across studies and confidence intervals show minimal overlap, while the I2 value exhibits moderate heterogeneity.
